# Bedside ocular ultrasonography for diagnosing increased intracranial pressure in patients with leptomeningeal metastases from non‐small‐cell lung cancer

**DOI:** 10.1002/cam4.5484

**Published:** 2022-12-04

**Authors:** Cheng Jiang, Yongjuan Lin, Huiying Li, Yu Xie, Tingting Yu, Jingyu Feng, Mingmin Huang, Aibin Guo, Haiyun Shen, YiDan Zhang, Zhenyu Yin

**Affiliations:** ^1^ Department of Geriatric Oncology Nanjing Drum Tower Hospital Affiliated of Nanjing University Medical School Nanjing China; ^2^ Department of Ultrasound Nanjing Drum Tower Hospital Affiliated of Nanjing University Medical School Nanjing China

**Keywords:** cerebrospinal fluid pressure, leptomeningeal metastases from non‐small‐cell lung cancer, ocular ultrasonography

## Abstract

**Objectives:**

To explore the diagnostic accuracy of ultrasound measurement of optic nerve sheath diameter (ONSD) and optic disc height (ODH) in detecting intracranial hypertension in non‐small‐cell lung cancer (NSCLC) patients with leptomeningeal metastases (LM).

**Methods:**

Seventy‐two patients with NSCLC‐LM and 65 patients with NSCLC were enrolled. The ONSD, ODH, eyeball transverse diameter (ETD), and eyeball vertical diameter (EVD) were measured by ultrasound. Subsequently, lumbar puncture was performed in NSCLC‐LM patients to measure cerebrospinal fluid pressure (CSFP), and intrathecal chemotherapy was regularly implemented. Pearson's correlation analysis was used to analyze the relationship between CSFP and ultrasound findings. The diagnostic accuracy of ONSD, ODH, and combined ONSD and ODH was evaluated by receiver operating characteristic (ROC) curve analysis and the corresponding area under the ROC curve (AUC).

**Results:**

The ONSD, ODH, ONSD/ETD, and ONSD/EVD values were higher in the NSCLC‐LM group (all *p* < 0.05). The ONSD, ODH, ONSD/ETD, and ONSD/EVD values were all elevated in the abnormally elevated CSFP group (all *p* < 0.05). ONSD, ODH, ONSD/ETD, and ONSD/EVD were positively correlated with CSFP (*r* = 0.531, 0.383, 0.534, and 0.535, all *p* < 0.0001). The AUCs for ONSD, ODH, ONSD/ETD, and ONSD/EVD to detect CSFP >280 mmH2O were 0.787 (95% CI: 0.64–0.93, sensitivity 68.75%, specificity 91.07%), 0.885 (95% CI: 0.81–0.96, sensitivity 100%, specificity 69.64%), 0.765 (95% CI: 0.64–0.89, sensitivity 81.25%, specificity 64.29%), and 0.788 (95% CI: 0.64–0.93, sensitivity 56.25%, specificity 91.07%), respectively. When ONSD was combined with ODH, the AUC was 0.913 (95% CI: 0.83–0.99, sensitivity 87.85%, specificity 85.70%). Furthermore, intrathecal chemotherapy was associated with a downtrend in CSFP and ultrasound findings.

**Conclusion:**

There are important advantages of using bedside ultrasonography for detecting elevated CSFP in NSCLC‐LM patients. Further research should be performed to evaluate the clinical significance of an enlarged ONSD and increased ODH in NSCLC‐LM.

## Graphical abstract

Bedside ocular ultrasonography is a noninvasive, bedside and easy‐to‐use way for detecting increased intracranial pressure in patients with leptomeningeal metastases from non‐small‐cell lung cancer. The measurement of combined ONSD and ODH may be a promising technique to detect elevated CSF pressure..

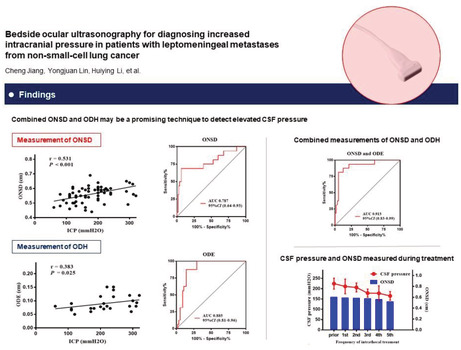

## 1 INTRODUCTION

In the advanced stage of non‐small‐cell lung cancer (NSCLC), 3%–4% of patients develop leptomeningeal metastasis (LM).^1^, ^2^ According to previous research, the median overall survival (OS) of NSCLC patients with LM is dismal, only 3 months.^3^ Timely diagnosis and treatment can improve the prognosis of patients. Lumbar puncture is a vital means for the diagnosis of meningeal metastasis, cerebrospinal fluid genomics, and intrathecal chemotherapy. However, NSCLC‐LM usually leads to intracranial hypertension, and lumbar puncture carries a high risk of cerebral hernia in such patients. Monitoring and management of intracranial pressure (ICP) is integral before lumbar puncture, and invasive ICP monitoring is the gold standard. However, the placement of invasive ventricular devices could be challenging due to the risk of infection and bleeding, lack of surgical availability, or high cost. Therefore, a noninvasive, bedside and easy‐to‐use detection tool is urgently needed to detect elevated ICP.

The optic nerve sheath (ONS), which surrounds the optic nerve, is separated by the subarachnoid space and filled with cerebrospinal fluid. The ONS is a continuation of the dura.^4^ When ICP is elevated, ONS is distensible in its retrobulbar segment.^5^ Moreover, prior reports have demonstrated that optic disc edema can be observed in chronic intracranial hypertension,^6^ which is due to swollen nerve fibers and the accumulation of extracellular fluid. US measurements of ONSD have been studied in different clinical settings and have shown a good correlation with invasive ICP values. These studies suggest that the ultrasonic measurement of ONSD is a reliable noninvasive method for assessing intracranial pressure.^7^, ^8^, ^9^ Under dynamic conditions, the correlation between ONSD and ICP remains valid.^10^ Of the studied ultrasound noninvasive ICP methods, ONSD, when compared with transcranial Doppler (TCD), showed higher accuracy in estimating ICP.^11^ However, a recent meta‐analysis showed that the optimal ONSD threshold varies greatly between studies.^4^, ^9^, ^12^, ^13^ Meanwhile, it is not clear whether ODH or ONSD combined with ODH can evaluate intracranial hypertension in diagnosing elevated intracranial hypertension. Previous studies have mainly focused on traumatic brain injury, cerebrovascular accidents, and central nervous system infection, but the application of ONSD ultrasound in lung cancer meningeal metastasis has not been reported.

Our main purpose was to recognize the accuracy of ultrasonographic measurements for the detection of cranial hypertension in patients with LM from NSCLC and to identify the optimal ONSD and ODH threshold.

## 2 MATERIALS AND METHODS

### 2.1 Ethics statement

We conducted a retrospective cohort study using data from the Department of Geriatric Oncology in Nanjing Drum tower Hospital in China. We signed informed consent with family members of all patients. This study was authorized by the Ethics Committee of Nanjing Drum tower Hospital. All procedures have proceeded in line with the ethical principles of the Declaration of Helsinki.

### 2.2 Patients

Between March 2021 and December 2021, 137 patients (72 with LM and 65 without LM) with NSCLC in the Department of Geriatric Oncology in Nanjing Drum tower Hospital were enrolled. All patients were diagnosed by clinical, CSF and radiographic findings based on the EANO–ESMO criteria.^14^ The inclusion criteria were as follows: (1) 18 years or older, (2) diagnosis of lung adenocarcinoma, and (3) patients with positive CSF cytology or with negative CSF cytology but with typical MRI and clinical symptoms requiring lumbar puncture and intrathecal chemotherapy. The exclusion criteria were as follows: (1) the use of medications that affect intracranial pressure in 1 week, (2) bilateral optic nerve involvement and preexisting ocular disease other than ametropia, and (3) recent use of mannitol and other drugs that affect intracranial pressure. The subjects in our control group were mainly postoperative adjuvant chemotherapy patients (pathological stage IIA‐IIIA), who had no neurological signs and symptoms and whose cerebrospinal metastases were ruled out by cranial and whole spine MRI.

### 2.3 Clinical data collection

**FIGURE 1:**
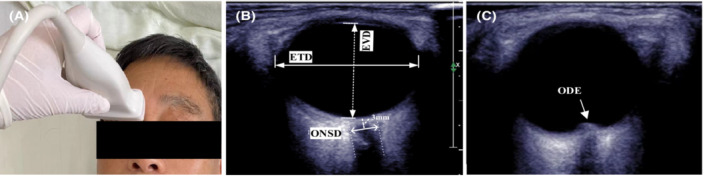
(A) Optic nerve ultrasound examination; (B) Measurement of ONSD, ETD, and EVD; An axial (transverse) view of the globe anteriorly, reveals the retrobulbar region with the optic nerve sheath (ONS) visible posteriorly. The optic nerve is visible within the ONS. (C) Measurement of ODH. ETD, eyeball transverse diameter; EVD, eyeball vertical diameter; ODE, optic disc edema; ONSD, optic nerve sheath diameter.

General information were recorded at admission: sex, age, BMI, oxyhemoglobin saturation, blood pressure, heart rate, and blood glucose. All patients were diagnosed by clinical symptoms, radiographic findings and cerebrospinal fluid cytology before enrollment. Two experienced ultrasound physicians performed ultrasound measurements at the bedside. Here, a 5‐ to 12‐MHz linear array ultrasonic probe from Philips iU22 (Philips Healthcare, USA) was used. The patient was supine, facing forward, and looking forward with the eyelids closed. A small volume of ultrasound gel is applied to the probe. And the probe was placed horizontally above the transverse axis of the eyeball with no pressure (Figure 1A). Slowly and vertically moved to the eyeball until the clearest and artifact‐free optimal plane was captured. The hypoechoic stripe posterior to the eyeball is the optic nerve, with sheaths on both sides visible and well‐defined. Images were chosen by two expert investigators together. The optic nerve sheath could be seen at the broadest part; within 1 mm from the eyeball, the optic nerve could be seen continuously for 6 mm without motion artifacts. Calipers were used to determine the point 3 mm posterior to the globe, and the ONSD of that point was measured in the vertical plane. The maximum diameter of the ETD (eyeball transverse diameter) and EVD (eyeball vertical diameter) on this plane were also measured (Figure 1B). Both eyes were measured three times, and the three values were averaged as the ONSD, ETD, and EVD of each eye. ODH was measured between the retina and the optic disc dome (Figure 1C).^15^ If no elevation of the optic disc was observed, ODH was recorded as 0 mm. The mean ONSD, ETD, EVD, and ODH of the patient were obtained by averaging the ONSD, ETD, EVD, and ODH of the bilateral eyes. The optic nerve ultrasound was performed about half an hour before the lumbar puncture in all patients in our enrollment. The optic nerve ultrasound took approximately 5 min to complete. Subsequently, the clinician performed a lumbar puncture in NSCLC‐LM patients, the CSFP was measured, and intrathecal chemotherapy was performed. The ultrasound operators and investigators were blinded to the patient's CSFP. We defined CSFP >280 mmH_2_O as abnormally elevated intracranial pressure.^16^ The CSFP was recorded by another clinical doctor.

### 2.4 Statistical methods

Use a *t*‐test (for data with a normal distribution) or Mann–Whitney *U*test (for data with a nonnormal distribution) for continuous variables when exploring the differences between NSCLC patients with and without LM and between LM patients with CSFP ≤280 mmH_2_O and CSFP >280 mmH_2_O. We implemented Pearson's correlation analysis to analyze the relationship between CSFP and optic nerve examinations. A receiver operating characteristic (ROC) curve was plotted to figure up the area under the ROC curve (AUC). All statistical analyses and graphics were performed with SPSS v26.0 (IBM) and GraphPad Prism v8.3.0 (GraphPad). A value of *p* < 0.05 (two‐sided) was regarded significant.

## 3 RESULTS

A total of 137 NSCLC patients were enrolled in our study, 72 of whom were identified with LM. The baseline characteristics and demographics of all patients are shown in Table 1. The ONSD measurements of patients without LM and with LM were 0.45 (0.43–0.48) cm and 0.57 (0.54–0.61) cm, respectively. No optic disc edema was present in patients without LM, while 33/72 (45.83%) patients with LM were observed to have optic disc edema. The average ONSD measurement and the proportion of optic disc edema in patients with LM were significantly increased compared with patients without LM (*p* < 0.05).

**TABLE 1:** Baseline variables and outcomes

Variables	LC without LM (n = 65)	LC with LM (n = 72)	*p* value
Female (%)	44 (67.69%)	53 (73.61%)	0.447
Age (years)	55.2 ± 7.34	53.96 ± 6.82	0.159
BMI (kg/m^2^)	21.81 ± 2.54	22.06 ± 3.03	0.128
Systolic blood pressure (mmHg)	121.19 ± 14.89	117.43 ± 17.11	0.142
Diastolic blood pressure (mmHg)	76.08 ± 9.18	75.82 ± 11.39	0.505
HR (bpm)	77.09 ± 10.88	79.93 ± 11.15	0.137
Blood glucose (mmol/L)	5.92 ± 1.22	5.93 ± 1.23	0.928
Oxyhemoglobin saturation (%)	97.12 ± 1.74	96.68 ± 2.03	0.171
Right‐ONSD (cm)	0.45 (0.40–0.49)	0.57 (0.53–0.61)	<0.001
Left‐ONSD (cm)	0.46 (0.41–0.49)	0.57 (0.54–0.60)	<0.001
Mean ONSD (cm)	0.45 (0.43–0.48)	0.57 (0.54–0.61)	<0.001
Right‐ETD (cm)	2.24 (2.15–2.32)	2.39 (2.29–2.45)	<0.001
Left‐ETD (cm)	2.27 (2.11–2.33)	2.35 (2.25–2.47)	<0.001
Mean ETD (cm)	2.23 (2.17–2.31)	2.36 (2.29–2.44)	0.526
Right‐EVD (cm)	2.01 (1.93–2.07)	2.00 (1.96–2.05)	0.771
Left‐EVD (cm)	1.98 (1.94–2.05)	2.00 (1.94–2.07)	0.182
Mean EVD	2.01 (1.95–2.04)	2.01 (1.96–2.05)	0.526
Right‐ONSD/R‐ETD	0.2 (0.18–0.22)	0.24 (0.22–0.26)	<0.001
Left‐ONSD/L‐ETD	0.21 (0.19–0.22)	0.24 (0.22–0.27)	<0.001
Right‐ONSD/R‐EVD	0.22 (0.21–0.24)	0.29 (0.26–0.30)	<0.001
Left‐ONSD/L‐EVD	0.23 (0.21–0.25)	0.29 (0.26–0.31)	<0.001
ODE (%)	0 (0%)	33 (45.83%)	<0.001

*Note*: Data are expressed as mean ± standard deviation, number (percentage), or median [interquartile range].

Abbreviation: ODE, optic disc edema.

Based on the results of lumbar puncture pressure, patients with LM were divided into a normal CSFP group (CSFP ≤280 mmH_2_O) and an abnormally elevated CSFP group (CSFP >280 mmH_2_O). The CSFP of the normal CSFP group (42 females, 14 males) and abnormally elevated CSFP group (11 females, five males) were 164.11 ± 35.84 mmH_2_O and 323.56 ± 22.41 mmH_2_O, respectively. The clinical characteristics, ONSD measurements and ODH are shown in Table 2. No significant differences were found between the two groups in sex, age, BMI, blood pressure, heart rate, blood glucose, oxyhemoglobin saturation, RANO, and PS scores (*p* > 0.05). The average ONSD measurements of the two groups were 0.57 (0.52–0.60) cm and 0.63 (0.57–0.66) cm. The ONSD measurements were higher in the abnormally elevated CSFP group (left: *p* < 0.001, right: *p* = 0.001, average: *p* < 0.001). In addition, the values of ONSD/ETD and ONSD/EVD were bilaterally higher in the abnormally elevated CSFP group (R‐ONSD/R‐ETD: *p* = 0.003, L‐ONSD/L‐ETD: *p* = 0.001; R‐ONSD/R‐EVD: *p* = 0.002; L‐ONSD/L‐EVD: *p* < 0.001). A total of 17/56 (30.36%, ODH range 0.00–0.07 cm) patients in the normal CSFP group had optic disc edema, while 33/72 (45.83%, ODH range 0.08–0.12 cm) patients in the abnormally elevated CSFP group had optic disc edema.

**TABLE 2:** The comparison of LM patients between CSFP≤280 mmH2O and CSFP >280 mmH2O

Demographic data	CSFP≤280 (*n* = 56)	CSFP>280 (*n* = 16)	*p* value
Female (%)	42 (75.00%)	11 (68.75)	0.617
Age (years)	53.59 ± 7.14	55.25 ± 5.58	0.394
BMI (kg/m^2^)	21.91 ± 3.31	22.75 ± 1.05	0.434
Systolic blood pressure (mmHg)	115.8 ± 17.75	123.97 ± 13.67	0.132
Diastolic blood pressure (mmHg)	75.55 ± 11.38	76.75 ± 11.72	0.714
HR (bpm)	80.41 ± 10.92	76.56 ± 11.79	0.226
Blood glucose (mmol/L)	5.99 ± 1.21	5.72 ± 1.33	0.451
Oxyhemoglobin saturation (%)	96.68 ± 2.02	96.69 ± 2.15	0.988
Admission RANO	6 (4–9)	8 (6–10.75)	0.056
Admission PS	2 (1–3)	3 (2–4)	0.058
Right‐ONSD (cm)	0.56 (0.51–0.60)	0.62 (0.57–0.65)	0.001
Left‐ONSD (cm)	0.56 (0.52–0.59)	0.64 (0.57–0.66)	<0.001
Mean ONSD (cm)	0.57 (0.52–0.60)	0.63 (0.57–0.66)	<0.001
Right‐ETD (cm)	2.35 (2.29–2.44)	2.41 (2.23–2.46)	0.77
Left‐ETD (cm)	2.35 (2.24–2.45)	2.36 (2.25–2.48)	0.775
Mean ETD (cm)	2.35 (2.31–2.44)	2.41 (2.23–2.45)	0.823
Right‐EVD (cm)	2.00 (1.96–2.05)	2.00 (1.93–2.06)	0.849
Left‐EVD (cm)	2.00 (1.95–2.07)	2.01 (1.94–2.05)	0.802
Mean EVD (cm)	2.01 (1.96–2.05)	2.01 (1.94–2.06)	0.765
Right‐ONSD/R‐ETD	0.24 (0.22–0.26)	0.26 (0.24–0.28)	0.003
Left‐ONSD/L‐ETD	0.24 (0.22–0.25)	0.27 (0.25–0.28)	0.001
Right‐ONSD/R‐EVD	0.29 (0.25–0.30)	0.31 (0.29–0.33)	0.002
Left‐ONSD/L‐EVD	0.28 (0.26–0.30)	0.32 (0.29–0.33)	<0.001
ODE (%)	17 (30.36%)	16 (100%)	<0.001
ODH (cm)	0.00 (0.00–0.07)	0.96 (0.08–0.12)	<0.001
CSFP (mmH2O)	164.11 ± 35.84	323.56 ± 22.41	<0.001

We divided patients with LM into two groups based on where papilledema was observed (Table 3). We found that the ONSD value, ONSD/ETD and ONSD/EVD were significantly higher in the group with papilledema than in the group without papilledema (*p* < 0.001, *p* = 0.012, *p* = 0.003). We also divided patients with LM into two groups based on whether they had symptoms of intracranial hypertension, whether MRI showed lateral ventricular widening, whether brain metastasis was present, whether MRI showed meningeal enhancement, and whether CSF had atypia cells. However, the ONSD value, ONSD/ETD and ONSD/EVD did not differ between the two groups.

**TABLE 3:** The comparison results of ONSD, ONSD/ETD, ONSD/EVD in different groups

	Cases (*n* = 72)	ONSD		ONSD/ETD		ONSD/EVD	
			*p*		*p*		*p*
Symptoms of raised CSFP			0.970		0.969		0.886
Yes	65	0.59 (0.54–0.61)		0.25 (0.22–0.27)		0.29 (0.27–0.30)	
No	7	0.57 (0.50–0.61)		0.24 (0.22–0.26)		0.29 (0.25–0.31)	
Lateral ventricle broadening by MRI			0.623		0.507		0.755
Yes	35	0.59 (0.54–0.61)		0.23 (0.22–0.26)		0.29 (0.27–0.30)	
No	37	0.57 (0.53–0.61)		0.24 (0.22–0.27)		0.29 (0.26–0.30)	
Accompanying brain metastasis			0.143		0.372		0.136
Yes	21	0.59 (0.56–0.62)		0.25 (0.24–0.26)		0.29 (0.28–0.31)	
No	51	0.57 (0.52–0.60)		0.24 (0.22–0.27)		0.28 (0.27–0.30)	
ODE			<0.001*		0.012*		0.003*
Yes	33	0.58 (0.56–0.62)		0.25 (0.24–0.27)		0.3 (0.28–0.31)	
No	39	0.55 (0.51–0.60)		0.23 (0.22–0.27)		0.28 (0.25–0.30)	
MRI finding at LM diagnosis			0.367		0.817		0.517
Yes	56	0.58 (0.55–0.61)		0.24 (0.23–0.27)		0.29 (0.27–0.30)	
No	16	0.55 (0.50–0.61)		0.24 (0.22–0.27)		0.29 (0.25–0.30)	
Positive for CSF cytology			0.106		0.132		0.197
Yes	56	0.58 (0.54–0.61)		0.24 (0.23–0.27)		0.29 (0.27–0.30)	
No	16	0.56 (0.50–0.59)		0.24 (0.21–0.26)		0.28 (0.25–0.30)	

* *p* < 0.05.

**FIGURE 2:**
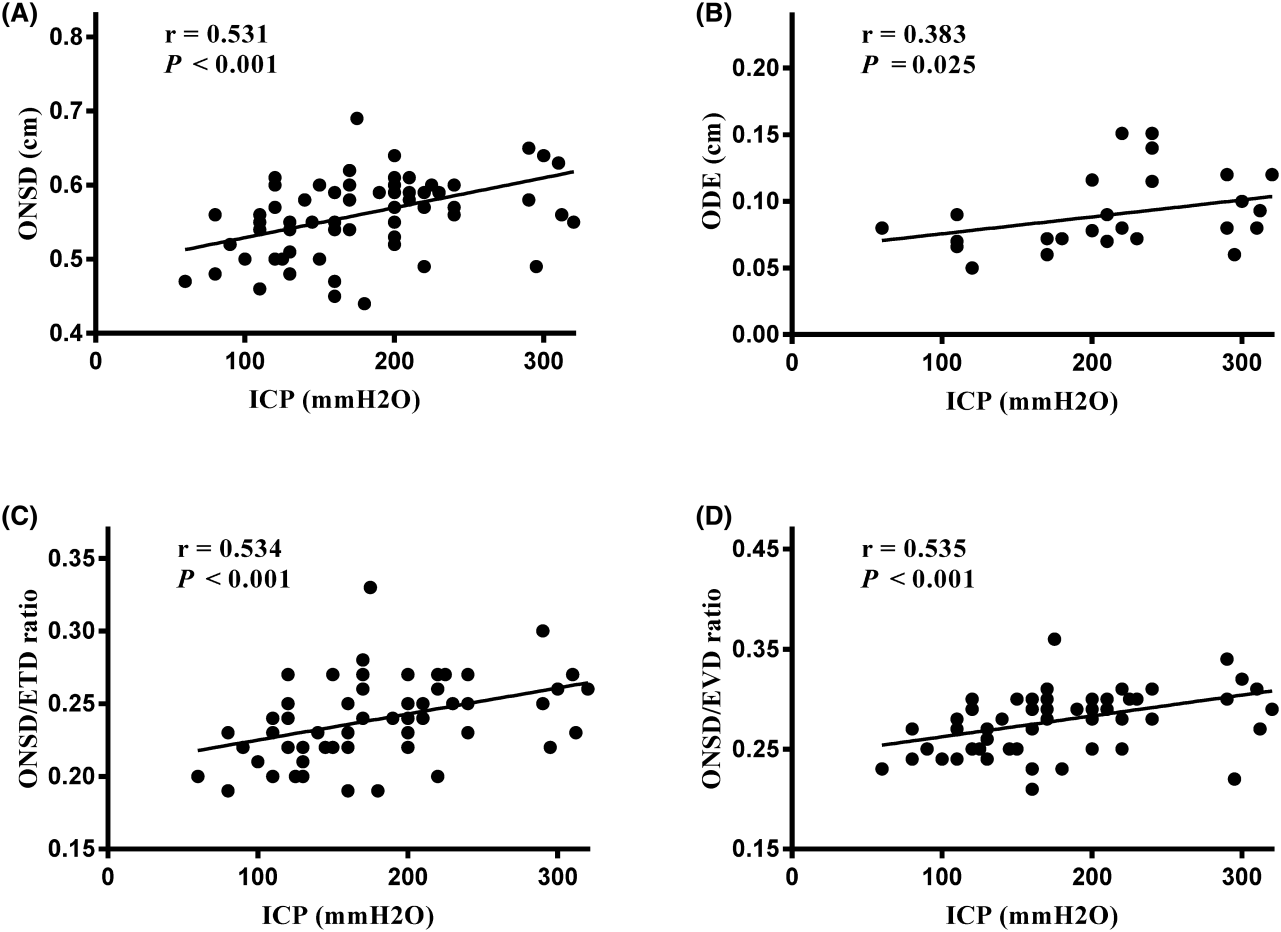
Pearson's test for the correlation of CSFP and optic nerve examination. (A) CSFP and ONSD. (B) CSFP and ODH. (C) CSFP and ONSD/ETD. (D) CSFP and ONSD/EVD.

According to the applied Pearson's correlation analysis, ONSD, ODH, ONSD/ETD, and ONSD/EVD were positively correlated with CSFP (*r* = 0.531, *p* < 0.001; *r* = 0.383, *p* = 0.025; *r* = 0.534, *p* < 0.001; *r* = 0.535, *p* < 0.001). Scatter diagrams are presented in Figure 2.

**FIGURE 3:**
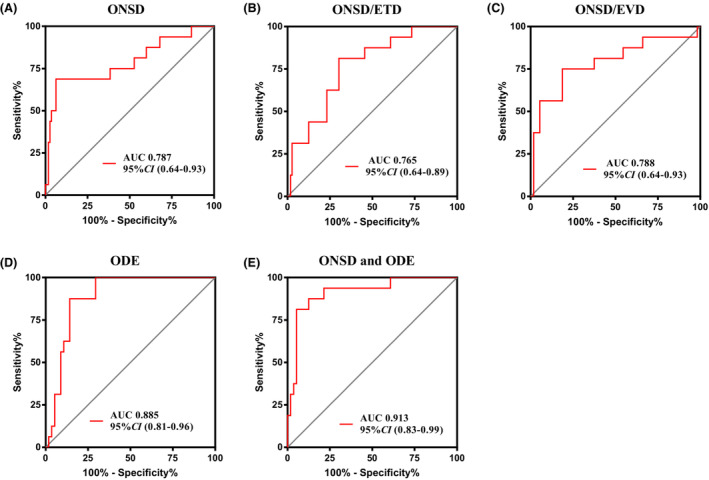
Receiver operating characteristic curves for analysis of diagnostic accuracy. (A) ONSD for the detection of CSFP greater than 280 mmH2O. (B) ONSD/ETD ratio for the detection of CSFP greater than 280 mmH2O. (C) ONSD/EVD ratio for the detection of CSFP greater than 280 mmH2O. (D) ODH for the detection of CSFP greater than 280 mmH2O. (E) Combined ONSD and ODH for the detection of CSFP greater than 280 mmH2O. AUC, area under the curve.

The ROC curve analysis found that the AUCs of ONSD, ONSD/ETD, ONSD/EVD, ODH, and combined ONSD and ODH in diagnosing abnormally elevated CSFP of NSCLC patients with LM were 0.787, 0.765, 0.788, 0.885, and 0.913, respectively. When the ONSD value was 0.615 cm, the AUC was 0.787, and the sensitivity and specificity were 68.75% and 91.07%, respectively. The ROC curves of ONSD/ETD, ONSD/EVD, ODH, combined ONSD and ODH are shown in Figure 3. The AUC was 0.765 (95% CI 0.64–0.89), 0.788 (95% CI 0.64–0.93), 0.885 (95% CI 0.81–0.96), and 0.913 (95% CI 0.83–0.99), and the corresponding threshold values were 0.765 (sensitivity of 81.25%, specificity of 64.29%), 0.305 (sensitivity of 56.25%, specificity of 91.07%), 0.055 cm (sensitivity of 100%, specificity of 69.64%), and 0.305 (sensitivity of 87.5%, specificity of 85.7%), respectively. See Table 4 and Figure 3 for details.

**TABLE 4:** ROC curve analysis of ONSD, ETD, EVD, and ODH for the detection of CSFP greater than 280 mmH2O

Influence factor	AUC	Cut‐off value	Sensitivity	Specificity	*p* value	95%CI	
						Lower limit	Upper limit
ONSD (cm)	0.787	0.615	68.75%	91.07%	<0.001	0.64	0.93
ONSD/ETD ratio	0.765	0.245	81.25%	64.29%	<0.001	0.64	0.89
ONSD/EVD ratio	0.788	0.305	56.25%	91.07%	<0.001	0.64	0.93
ODH (cm)	0.885	0.055	100%	69.64%	<0.001	0.81	0.96
ONSD + ODH	0.913	‐	87.50%	85.70%	<0.001	0.83	0.99

**FIGURE 4:**
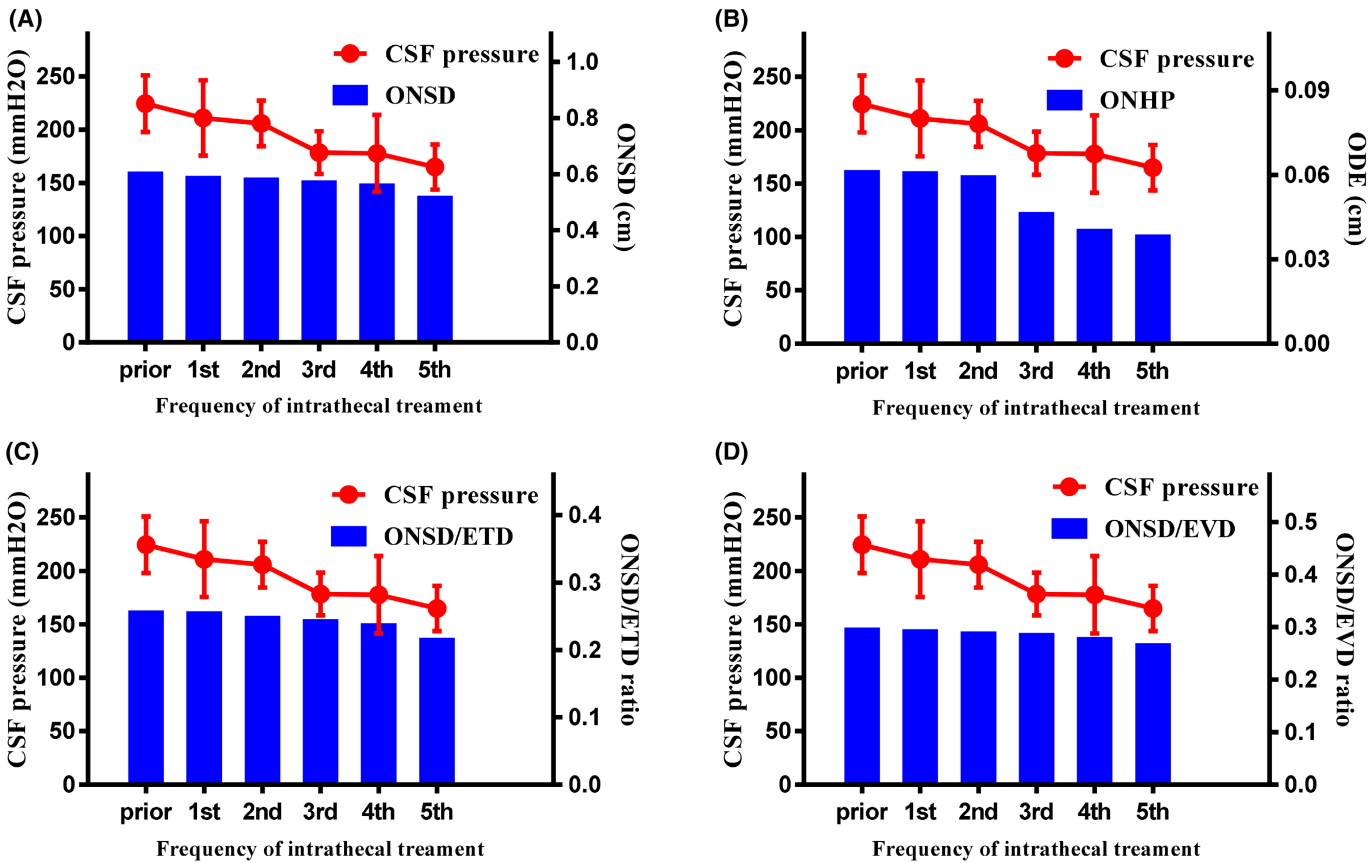
CSFP and optic nerve values measured prior to each treatment in NSCLC‐LM patients. (A) CSFP and ONSD. (B) CSFP and ODH. (C) CSFP and ONSD/ETD. (D) CSFP and ONSD/EVD.

**FIGURE 5:**
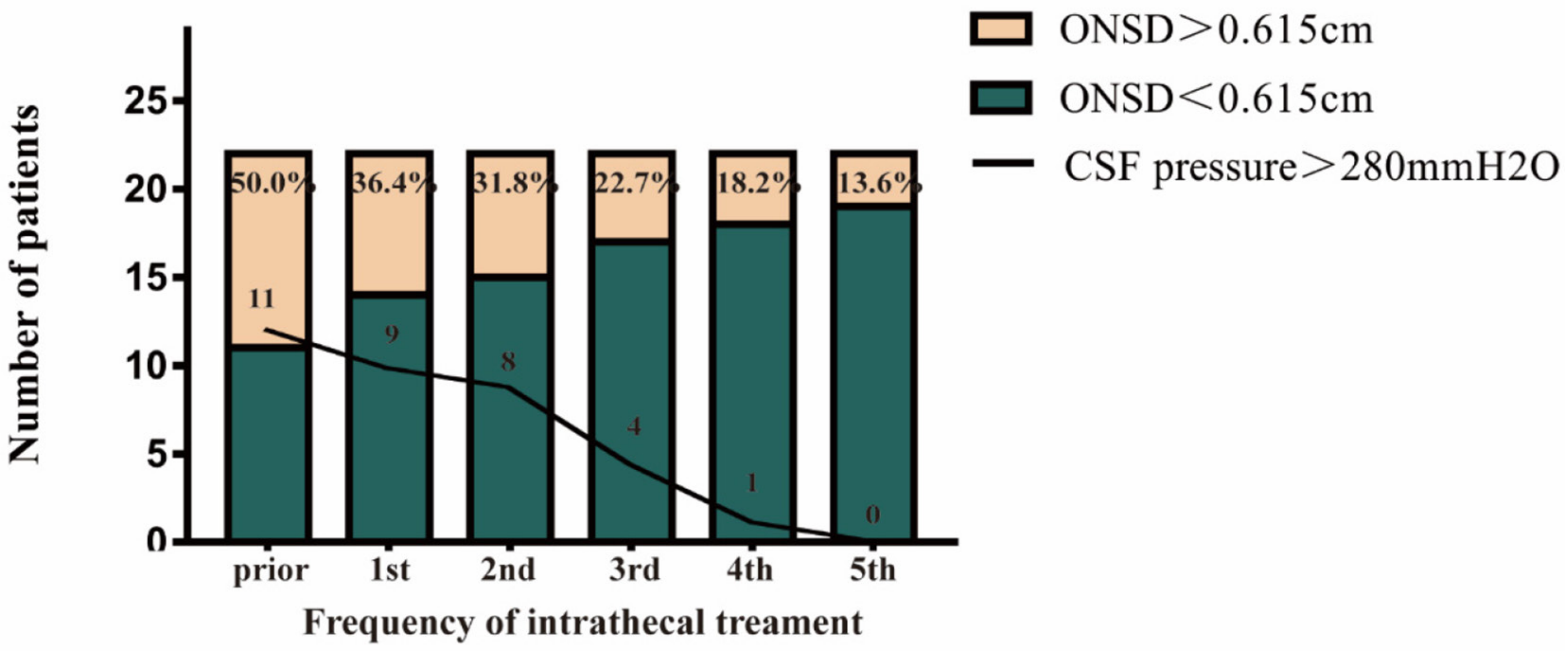
The changing of CSFP and proportion of NSCLC‐LM patients.

Twenty‐two patients with LM received six intrathecal chemotherapies. Intrathecal pemetrexed at a dose of 30 mg was administered every 21 days via lumbar puncture on days 1 and 8.^17^ Half of them (50%; *n* = 11/22) showed a baseline ONSD>0.615 cm, and of the 22 patients, a baseline CSFP>280 mmH_2_O was present in 11 patients (11/22; 50%) on admission. We discovered that the initiation of intrathecal chemotherapy was associated with a downtrend in CSFP and ultrasound findings, including ONSD, ODH, ONSD/ETD, and ONSD/EVD (Figure 4). There was an apparent decline in the proportion of patients with widened ONSD and in the number of patients with abnormally elevated CSFP following each intrathecal chemotherapy (Figure 5). From a baseline value of 50%, the proportion of patients whose ONSD was >0.615 cm decreased to 31.8% after the second intrathecal chemotherapy and to 13.6% after the fifth intrathecal chemotherapy. From a baseline value of 11, the number of patients whose CSFP >280 mmH_2_O decreased to eight after the second intrathecal chemotherapy and to zero after the fifth intrathecal chemotherapy.

## 4 DISCUSSION

NSCLC‐LM often leads to an elevation of intracranial pressure, which could severely disrupt nerve function and possibly even lead to a cerebral hernia, squeezing important structures and inducing coma or death in severe cases. Early recognition and management can improve the outcomes of LM patients.^18^ However, invasive ICP monitoring is the “gold standard”.^19^ Rapid and bedside identification of NSCLC‐LM patients requiring rapid control of ICP may drive effective management in an urgent or resource‐constrained environment.^12^

In previous studies, researchers have assessed only ONSD as a monitor for intracranial hypertension; now, we demonstrate additional optic nerve abnormalities with optic disc elevation. Our results indicate that ultrasonic ONSD and ODH are strongly related to CSFP. With the decrease in CSF pressure, ONSD also showed a downward trend. The measurement of ONSD and ODH could be used to detect abnormally increased CSFP in NSCLC‐LM patients. This is the first time we have used optic nerve ultrasound to predict CSFP associated with metastatic meningeal carcinoma.

There have been some studies on ultrasonic measurement of ONSD for the diagnosis of high ICP,^20^, ^21^ while there are relatively few studies on the measurement of ODH for the diagnosis of high ICP. Rajajee V et al. compared the accuracy of optic nerve ultrasound in assessing intracranial pressure with sharp fluctuations and stability. They found that the specificity and positive predictive value of ONSD measurement prominently reduced in the setting of acutely ICP fluctuation because of the delayed reversal of ONS dilation.^22^ Hayreh SS et al. explored the pathogenesis of optic disc edema by performing basic, experimental, and clinical studies. They found that acutely elevated CSFP did not produce optic disc edema, while chronically elevated CSFP can lead to optic disc edema.^6^ Therefore, ONSD and ODH are of great value in assessing CSFP in NSCLC patients with meningeal metastasis, which is subacute or chronic. In our study, ONSD and ODH were positively correlated with CSFP. ONSD values significantly increased in NSCLC‐LM groups, especially in patients with CSF pressure greater than 280 mmH2O. More optic disc edema was available in the group with abnormally high CSFP. These discoveries confirmed the biological relationship between ONSD, ODH, and CSFP. However, in clinical practice, the presence of a biological correlation does not automatically translate to satisfactory diagnostic accuracy. As ONSD and ODH are the most potential noninvasive monitors to recognize patients requiring empirical treatment, high sensitivity is foremost. Meanwhile, a test with high specificity can be superior to routine clinical evaluation and is likely to outperform invasive testing. In our study, the optimal threshold of ONSD obtained only a sensitivity of 68.75% (along with a minimal specificity of 91.07%), which could not meet our predetermined minimum criteria for acceptable accuracy. With this threshold, nearly one‐third of patients with truly intracranial hypertension would be suffering from error classification, which is unacceptable given the severe consequences of elevated intracranial pressure if left untreated. In addition, ODH achieved an AUC greater than 0.8 for the detection of concurrent CSF pressure greater than 280 mmH_2_O, but the optimal threshold of ODH achieved a specificity of 69.64% (in conjunction with minimal sensitivity 100%), which means that nearly one‐third of patients diagnosed with elevated ICP using this ODH threshold will, in fact, not have intracranial hypertension and may be exposed to the risks of overtreatment. ONSD and ODH both performed badly in recognizing patients requiring an aggressive therapy to treat intracranial hypertension. However, when ONSD was combined with ODH, the optimal threshold achieved a specificity of 85.7%, in conjunction with a sensitivity of 87.5%. Therefore, we could use the combined ONSD and ODH to detect abnormally elevated CSF pressure in NSCLC‐LM patients.

A recent study suggests that the extent to which ONS swell with increased intracranial pressure may vary from person to person.^23^ Therefore, to increase the accuracy of the optic nerve ultrasound method, ONSD/ETD and ONSD/EVD were measured to evaluate their relationship with CSF pressure. Our study suggested that these variables may be utilized as a possible alternative, which is consistent with other studies.^16^, ^21^, ^24^ However, we still need more investigations before using the ONSD/ETD ratio and other variables for clinical decision‐making. Our study has several strengths. This is the first time we have used ultrasound to assess CSF pressure in NSCLC‐LM patients. This is the only study to assess the ability of combined ONSD and ODH to identify patients requiring timely treatment of abnormally elevated ICP. Continuous changes in ultrasound findings and CSF pressure were observed before and after treatment. Most importantly, sonographers remain completely and rigorously blinded to the CSF pressure and clinical details of the patients. The standards for reporting diagnostic accuracy recognize blinding as a vital element of high‐quality diagnostic studies.^25^

There are still several limitations in our study. A larger sample size may have narrowed the CIs in the estimates of accuracy—there were only 16 (22.22%) CSF pressure > 280 mmH2O in the analysis. The ECOG score for all patients was between 0 and 2; therefore, the most severe cases were likely excluded. Moreover, we compared ultrasound values with lumbar CSF pressure instead of invasive ICP measurement, yet there may be differences in the CSF pressure between intracranial and spinal compartments.^26^

In conclusion, our results demonstrated a significant relationship between ultrasound findings and CSF pressure. The measurement of combined ONSD and ODH may be a promising technique to detect elevated CSF pressure. A prospective multicenter clinical trial with a larger cohort is necessary to further evaluate the clinical significance of an enlarged ONSD and increased ODH in NSCLC‐LM.

## AUTHOR CONTRIBUTIONS

**Cheng Jiang:** Investigation (equal); writing – original draft (lead); writing – review and editing (lead). **Yongjuan Lin:** Data curation (equal); software (lead). **Huiying Li:** Formal analysis (equal); supervision (equal); validation (equal). **Yu Xie:** Investigation (equal); methodology (equal); resources (equal). **Tingting Yu:** Funding acquisition (equal); project administration (equal); writing – original draft (supporting); writing – review and editing (supporting). **Jingyu Feng:** Writing – original draft (supporting); writing – review and editing (supporting). **Mingmin Huang:** Funding acquisition (supporting); investigation (supporting); methodology (supporting); project administration (supporting). **Aibin Guo:** Resources (supporting); supervision (supporting); validation (supporting). **Haiyun Shen:** Data curation (equal); formal analysis (supporting); methodology (equal); software (supporting). **Yidan Zhang:** Data curation (equal); formal analysis (equal); methodology (equal). **Zhenyu Yin:** Conceptualization (lead).

## FUNDING INFORMATION

This work was supported by funding from the Medical Key Science and Technology Development Project of Nanjing (No. ZKX18014), the Cadre Health Care Project of Jiangsu Province (No. BJ18006, BJ19001, BJ21002), and the Cancer Research Funding of CSCO‐Hausen (No. Y‐HS2019‐5).

## CONFLICT OF INTEREST

All authors have no conflicts of interest to disclose.

## Data Availability

Data openly available in a public repository.

## References

[1] Li YS , Jiang BY , Yang JJ , et al. Leptomeningeal metastases in patients with NSCLC with EGFR mutations. J Thorac Oncol. 2016;11(11):1962‐1969. 27539328 10.1016/j.jtho.2016.06.029

[2] Yang JCH , Kim SW , Kim DW , et al. Osimertinib in patients with epidermal growth factor receptor mutation‐positive non‐small‐cell lung cancer and leptomeningeal metastases: the BLOOM study. J Clin Oncol. 2020;38(6):538‐547. 31809241 10.1200/JCO.19.00457 PMC7030895

[3] Xu Y , Hu M , Zhang M , et al. Prospective study revealed prognostic significance of responses in leptomeningeal metastasis and clinical value of cerebrospinal fluid‐based liquid biopsy. Lung Cancer. 2018;125:142‐149. 30429013 10.1016/j.lungcan.2018.08.017

[4] Agrawal D , Raghavendran K , Zhao L , Rajajee V . A prospective study of optic nerve ultrasound for the detection of elevated intracranial pressure in severe traumatic brain injury. Crit Care Med. 2020;48:e1278‐e1285. 33048902 10.1097/CCM.0000000000004689 PMC7708401

[5] Geeraerts T , Merceron S , Benhamou D , Vigue B , Duranteau J . Noninvasive assessment of intracranial pressure using ocular sonography in neurocritical care patients. Crit Care. 2008;12(Suppl 2):P117. 10.1007/s00134-008-1149-x 18509619

[6] Hayreh SS . Pathogenesis of optic disc edema in raised intracranial pressure. Prog Retin Eye Res. 2016;50:108‐144. 26453995 10.1016/j.preteyeres.2015.10.001 PMC4698254

[7] Robba C , Santori G , Czosnyka M , et al. Optic nerve sheath diameter measured sonographically as non‐invasive estimator of intracranial pressure: a systematic review and meta‐analysis. Intensive Care Med. 2018;44:1284‐1294. 30019201 10.1007/s00134-018-5305-7

[8] Wang LJ , Chen LM , Chen Y , et al. Ultrasonography assessments of optic nerve sheath diameter as a noninvasive and dynamic method of detecting changes in intracranial pressure. JAMA Ophthalmol. 2018;136(3):250‐256. 29392301 10.1001/jamaophthalmol.2017.6560 PMC5885896

[9] Dubourg J , Javouhey E , Geeraerts T , Messerer M , Kassai B . Ultrasonography of optic nerve sheath diameter for detection of raised intracranial pressure: a systematic review and meta‐analysis. Intensive Care Med. 2011;37(7):1059‐1068. 21505900 10.1007/s00134-011-2224-2

[10] Launey Y , Nesseler N , Le Maguet P , Mallédant Y , Seguin P . Effect of osmotherapy on optic nerve sheath diameter in patients with increased intracranial pressure. J Neurotrauma. 2014;31(10):984‐988. 24372319 10.1089/neu.2012.2829

[11] Chiara R , Danilo C , Tamara T , et al. Ultrasound non‐invasive measurement of intracranial pressure in neurointensive care: a prospective observational study. PLoS Med. 2017;14(7):e1002356. 28742869 10.1371/journal.pmed.1002356 PMC5526499

[12] Donovan J , Oanh P , Dobbs N , Phu NH , Thwaites GE . Optic nerve sheath ultrasound for the detection and monitoring of raised intracranial pressure in tuberculous meningitis. Clin Infect Dis. 2021;73(9):e3536‐e3544. 33283229 10.1093/cid/ciaa1823 PMC8563195

[13] Robba C , Pozzebon S , Moro B , Vincent JL , Taccone FS . Multimodal non‐invasive assessment of intracranial hypertension: an observational study. Crit Care. 2020;24(1):379. 32591024 10.1186/s13054-020-03105-z PMC7318399

[14] Le Rhun E , Weller M , Brandsma D , et al. EANO‐ESMO clinical practice guidelines for diagnosis, treatment and follow‐up of patients with leptomeningeal metastasis from solid tumours. Ann Oncol. 2017;28(suppl_4):iv84‐iv99. 28881917 10.1093/annonc/mdx221

[15] Tamburrelli C , Salgarello T , Caputo CG , Giudiceandrea A , Scullica L . Ultrasonographic evaluation of optic disc swelling: comparison with CSLO in idiopathic intracranial hypertension. Invest Ophthalmol Vis Sci. 2000;41(10):2960‐2966. 10967051

[16] Du J , Deng Y , Li H , et al. Ratio of optic nerve sheath diameter to eyeball transverse diameter by ultrasound can predict intracranial hypertension in traumatic brain injury patients: a prospective study. Neurocrit Care. 2020;32(2):478‐485. 31218637 10.1007/s12028-019-00762-z

[17] Fan C , Zhao Q , Li L , et al. Efficacy and safety of intrathecal Pemetrexed combined with dexamethasone for treating tyrosine kinase inhibitor‐failed leptomeningeal metastases from EGFR‐mutant NSCLC‐a prospective, open‐label, single‐arm phase 1/2 clinical trial (unique identifier: ChiCTR1800016615). J Thorac Oncol. 2021;16(8):1359‐1368. 33989780 10.1016/j.jtho.2021.04.018

[18] Kwan K , Ullman JS , Schneider J , Hawryluk G , Ghajar J . Guidelines for the Management of Severe Traumatic Brain Injury: Recommendations from the 2017. 4th ed.; 2018.

[19] Geeraerts T , Launey Y , Martin L , et al. Ultrasonography of the optic nerve sheath may be useful for detecting raised intracranial pressure after severe brain injury. Intensive Care Med. 2007;33(10):1704‐1711. 17668184 10.1007/s00134-007-0797-6

[20] Beyaz SG , Kaya B , Ulgen AM , Sahin F , Kocayigit H , Issi ZT . Evaluation of increased intracranial pressure with the optic nerve sheath diameter by ultrasound in Epiduroscopic neural laser discectomy procedures. Pain Physician. 2021;24(5):E595‐E600. 34323446

[21] Şık N , Erbaş İM , Demir K , Yılmaz D , Duman M . Bedside sonographic measurements of optic nerve sheath diameter in children with diabetic ketoacidosis. Pediatr Diabetes. 2021;22(4):618‐624. 33538381 10.1111/pedi.13188

[22] Rajajee V , Fletcher JJ , Rochlen LR , Jacobs TL . Comparison of accuracy of optic nerve ultrasound for the detection of intracranial hypertension in the setting of acutely fluctuating vs stable intracranial pressure: post‐hoc analysis of data from a prospective, blinded single center study. Crit Care. 2012;16(3):R79. 22578001 10.1186/CC11336 PMC3580621

[23] Kerscher SR , Schöni D , Neunhoeffer F , et al. The relation of optic nerve sheath diameter (ONSD) and intracranial pressure (ICP) in pediatric neurosurgery practice‐part II: influence of wakefulness, method of ICP measurement, intra‐individual ONSD‐ICP correlation and changes after therapy. Child's Nerv Syst. 2020;36(1):107‐115. 31392457 10.1007/s00381-019-04336-4

[24] Kerscher SR , Zipfel J , Groeschel S , Bevot A , Haas‐Lude K , Schuhmann MU . Comparison of B‐scan ultrasound and MRI‐based optic nerve sheath diameter (ONSD) measurements in children. Pediatr Neurol. 2021;124:15‐20. 34508997 10.1016/j.pediatrneurol.2021.08.002

[25] Bossuyt PM , Reitsma JB , Bruns DE , et al. STARD 2015: an updated list of essential items for reporting diagnostic accuracy studies. BMJ. 2015;351:h5527. 26511519 10.1136/bmj.h5527 PMC4623764

[26] Beare NA , Glover SJ , Lewallen S , et al. Prevalence of raised intracranial pressure in cerebral malaria detected by optic nerve sheath ultrasound. Am J Trop Med Hyg. 2012;87(6):985‐988. 23033398 10.4269/ajtmh.2012.11-0459 PMC3516101

